# Inquiry into the Influence of Chemistry on the Operation of Animal Bodies

**Published:** 1802-03

**Authors:** 

**Affiliations:** Physician at the Hague


*Inquiry into the Influence of Chemistry on the Operation of Animal Bodies.*

*An abridged Translation of a Treatise written on that Subject in the Dutch Language, by* Dr. ONTYD, *Physician at the Hague.*

AMONG the different Arts and Sciences, in which considerable progress has been made during the last century, we may doubtless place Chemistry in the first rank. What an astonishing difference do we find in the state of our knowledge respecting this important science, between the commencement and the close of the last age! That we are much better acquainted with the component parts of natural bodies, the changes that take place in consequence of their union and influence upon each other, and the operation of chemical laws upon animal bodies, than our ancestors were, is a fact that no one can dispute: Therefore, it is not surprising that, in the present day, frequent attempts should be made to render the discoveries in this

---

this important ſcience ſubſervient to the different branches of medicine, and employ them to explain various appearances in animated nature; nay, there are ſome who have ventured to attribute the firſt principles both of inanimate and animated bodies, and all the appearances of the latter, to the union and affinities of the different materials of which animated bodies are compoſed. Reil, who, with Galvani, may be conſidered as the founder of this new chemical ſect, propoſed his ſyſtem with a degree of caution, and ſimply maintained, that moſt of the appearances in living bodies were principally to be aſcribed to the chemical properties of animated matter. He does not attempt to apply his hypotheſis to the practice of the healing art, but ingenuouſly confeſſes, that ſince we are as yet ignorant in what manner the mixtures in organized bodies are changed by their action upon them, we ought not to truſt to any theories in the cure of diſeaſes, but prefer that mode of practice which experience has diſcovered to be efficacious.

But this cautious manner is not equally apparent in the writings of his followers; they not only aſcribe every appearance in living bodies, without exception, to ſome chemical cauſe, but they attempt to explain the nature of diſeaſes, and introduce remedies, upon the principles of chemiſtry alone. Thus Girtanner, Beddoes, Cruikſhanks, and others, maintain, that the venereal diſeaſe owes its origin to a deficiency of the acidifying principle (oxygen), and attempt the cure by ſupplying this defect. Dr. Trotter aſcribes the dyſentery to the ſame cauſe. Dr. Rollo proceeds ſo far, as to aſcribe idiopathic diſeaſes to a ſuperabundance or a defect of oxygen: He has accordingly formed a conciſe Materia Medica, by means of which, according to his ſyſtem, a *plus* or *minus* of oxygen may be adapted to the particular nature of the diſeaſe; and he has lately publiſhed, by order of the King of Pruſſia, a *chemical mode* of curing fevers. Profeſſor Reich alſo preſents us with a ſure remedy to cure every ſpecies of fever very expeditiouſly; for as all fevers, according to him, proceed from a leſſer or greater defect in the requiſite quantity of acid matter, thus are they to be cured by the ſame kind of remedy, which conſiſts of the plentiful uſe of acids.

But, notwithſtanding a prudent application of our chemical knowledge to the human œconomy promiſes great advantages both to the theory and practice of phyſic; notwithſtanding that the diſcovery of a new remedy, calculated to alleviate the miſeries of mankind, is moſt deſirable to every humane practitioner; yet, on the other hand, diſcertion requires that no novel remedy ſhould approach a ſick-bed, until we have fully inveſtigated the principles upon which its boaſted efficacy is founded, and and the reports of its advantageous application have been ascertained to be genuine. Such precautions become peculiarly neceffary at a period when foreign practitioners crowd upon us fuch numbers of new theories, and new modes of practice, of which the majority are found by experience to merit our contempt.

Thefe confiderations have prompted me to examine how far the actions of animal bodies, either in a healthy or difeafed ftate, are limited by the Laws of Chemiftry; I fhall confider this queftion both as it relates to Phyfiology and Pathology.

Firft, I fhall endeavour to trace to what extent the motions of animal bodies in a healthy ftate are influenced by chemical laws; and then, fecondly, enquire into the degree of influence thefe laws exert, either in increafing or in changing the nature of the difeafes to which animal bodies are fubject, and examine from facts, what affiftance chemiftry has hitherto afforded us, to difcover the nature of difeafes, or the methods of cure.

As no one among the fupporters of this new fyftem has advanced farther in the application of chemical laws to the Phyfiology of the human body than Prof. Reich, the firft paragraph of his fyftem fhall ferve us as a clue to direct our enquiries.

The Profeffor begins his Treatife in this manner: "Every one who views with open eyes, and unprejudiced mind, the appearances in the human body, will feel himfelf compelled to acknowledge, that all its motions of every kind, are the refult of an uninterrupted chain in the operations of animal chemiftry, whereby a conftant change is produced in the mixture of organic matter. Thefe animo-chemical operations are the effects of an inceffant counteraction of oppofite powers or principles againft each other, without which, nothing throughout all fpace can exift, or be fuppofed to exift. The perpetual compofitions, changes, feparations, of different materials, their mutual correfpondence refpecting quantities and qualities, the difference of the various organs in which thefe changes are produced, and their fpecific actions, muft inevitably occafion a great diverfity of effects."

Thefe ftrong affertions are totally unfupported, either by argument or facts. He affumes it as an indubitable axiom, that all animal functions are founded on the mixture of organic matter, and builds upon this all his fubfequent aphorifms, which are in fact no other than inductions from his firft propofition. Whether thefe be fo obvious to every unprejudiced eye, we fhall leave to the judgement of our Readers. We are not afhamed to acknowledge the great imperfection of our own optics, and to declare openly, that the whole of what Reil has advanced from the writings of Humbolt and Ritter, whence he has has obviously borrowed his ſyſtem of fevers, has not been ſufficiently efficacious to diſpel the miſt from our eyes. It ſtill remains extremely doubtful with us, whether the firſt principle or cauſe of vitality, is to be found in any particular combination of animal matter.

Notwithſtanding the full conviction we entertain of the advantages which may be derived to the different branches of medicine by the diſcreet application of chemical ſcience to the animal œconomy; notwithſtanding we freely acknowledge that many appearances in organized bodies are happily explained by the application of chemical principles, yet we cannot admit the aſſertions of Reil, Reich, Schelver, and others, that all the operations of the human body are derived from particular combinations of organized matter, and that the vital power itſelf is merely an effect of the organization and peculiar combinations of animal matter. It appears to us that, in this caſe alſo, the medium is the ſafeſt road: For though we admit that theſe have a powerful influence in the various operations of animated bodies, nay, that they contribute to the ſupport of the vital powers, and that changes in the one are frequently productive of changes in the other, yet we cannot allow that ſimple organization is the baſis and cauſe of every appearance. The *vital power* appears to us totally different from *organization*; at leaſt this opinion appears much more favourable to the explanation of the phenomena in animated bodies. Let us but compare *dead* with *living* bodies, and what an aſtoniſhing difference preſents itſelf. In unwrought nature, matter is paſſive; and left entirely to itſelf, is not ſubject to the leaſt change. Whenever it is brought into action by ſome external cauſe, this action perpetually decreaſes, until it is totally ſtopt; it returns gradually from the ſtate of activity to which it was compelled, to a ſtate of reſt, in which it remains.

Whenever one maſs of matter is made to act upon another, the one loſes its motive power in exact proportion to the degree in which the other partakes of it; ſo that the quantity of motion excited, ſolely depends upon the quantity of power received.

When a chemical combination takes place between two ſubſtances, they both loſe by this union, ſome, if not *all*, of thoſe properties they ſeparately poſſeſſed, and form a new ſubſtance different from either.

This loſs of motion, and this change of properties in dead matter, are perfectly uniform and ſubject to the general laws of Nature. The laws of mechanic motion regulate the firſt, thoſe of chemiſtry the laſt; ſo that an experienced mechanic, or chemiſt, when they can depend upon the goodneſs of their materials, als, will always be able to know, *a priori*, what will be the refult of their experiments.

But in living bodies, the cafe is far otherwife. Organized matter, it is true, may experience a diminution of motion, but not complete reft. The vital actions of the organs themfelves, the perpetual changes that take place in their conftituent parts, the inceffant action of different ftimuli upon organized bodies, banifh entirely the idea of perfect reft.

The common properties of living fubftances differ effentially from thofe of inanimate matter. The changes which the firft continually experience, the different capacities and powers poffeffed by the numberlefs living beings which fill the univerfe, and thofe particularly which relate to their forms and modes of exiftence, plainly manifeft that they are governed by their own exclufive laws, depending upon the clafs to which they belong; and that the organized matter of which they are compofed, has very different properties from thofe belonging to dead matter in its natural ftate.

Living bodies, in a ftate of health, are in conftant motion, and are endowed with the power of refifting, to a certain degree, the mechanic and chemical actions of inanimate bodies, as alfo the properties of changing *dead* matter into *organized and living*. They poffefs the power in a greater or lefs degree, of deftroying the fenfible properties of the fubftances expofed to their action, while they preferve their own unimpaired. They protect the animal machine from diffolution and corruption, by acting upon foreign fubftances, and changing them into their own natures.

Thefe various fubftances acquire peculiarities very different from thofe they originally poffeffed. Saline bodies, for example, whether they be alkaline or acid, as foon as they have undergone the operation of digeftion in the ftomach of a living animal, immediately lofe their former properties and chemical characteriftics. The food, whether it be taken from the animal or vegetable kingdom, lofes every trace of its original ftate, and becomes prepared for being affimilated to the nature of the living body; being tranfmuted into a chyle of one uniform nature, however different the fubftances might be from which it was prepared.

But, although they are thus affimilated by the digeftive powers, yet thefe various fubftances differ materially from each other, refpecting their adaption to be transformed into the nature of the animals which confume them; and hence it is, that different animals require different kinds of food, which is alfo the cafe with the fame animal at different periods.

This diverfity in fubftances which are expofed to the action of of living beings, cannot be the effect of a chemical caufe; for then would the change produced in the food be conftant and uniform; and the chyle, inftead of being a fluid of fimilar properties, would poffefs thofe of the nutritive fubftances from whence it was taken.

Digeftion itfelf is not a chemical but a vital operation, by virtue of which all organized beings are endowed with the power of changing thefe different foods into their own nature, until it finally affumes the form and properties of that animal by whofe organs it was changed. Meat and bread, after they have gone through the procefs of digeftion in a Man or a Dog, are transformed into the fpecific nature of each. Thus we fee numberlefs plants, nourifhed by the fame water and the fame air, affume very different organized forms, which can only be afcribed to a peculiar living power inherent in each.

The effects are totally different whenever difeafe, or any other caufe, deftroys the power of living bodies to affimilate fubftances to their own nature. The food, in fuch cafes, either retains its particular properties, or it undergoes the fame changes *in* the body to which it was liable *out* of it. Animal and vegetable bodies, inftead of forming of chyle, run into an acid or a putrid fermentation. Saline fubftances alfo obferve the laws of chemiftry, uniting with their correfponding falts. This is illuftrated by the effects of magnefia upon acidities in the *primæ viæ*. Whenever the fubftances taken into the body poffefs great chemical powers, as the concentrated acids for example, and cauftic alkalies, they deftroy, by the violence of their ftimulus, the organic ftructure, form a chemical union with its component parts, with which they have an immediate attraction, and it becomes a dead unorganized mafs.

How great, therefore, is the difference between dead and animated matter! The firft is, in its own nature, *passive*, fimply poffeffing the power of attraction and repulfion; the other is in perpetual action, and produces perpetual changes! "This fummit of the Alps, (exclaims the ingenious Ith) thefe vales, and plains, and lakes, remain in the fame ftate as they were in the days of our remoteft anceftors; yet organized beings live, move, and change inceffantly: The young become old, the fmall grow large, one generation arifes, and preffes upon another. Nothing remains inert, an inceffant production and diffolution take place, of beings which, from the firft moment of their exiftence to that of their decay, maintain an uninterrupted courfe of changes and transformations.

Moreover, mechanic action produces very different effects in dead and in living bodies. Machinery is utterly unable to fupport itfelf in a ftate of uniform action, to fupply the loffes it fuftains by friction, or repair the injuries to which it may be occafionally occaſionally expoſed. The current of a river wears away its banks; but the action of the fluids in the living body, ſupplies defects, and increaſes the firmneſs of the veſſels through which it circulates. The ſame remark is applicable to chemical powers. Doubtleſs, chemical operations and affinities take place in animal bodies; nay, the experiments made by Galvani, Humbold, and Ritter, render it very probable that all the functions of life are cloſely connected with chemical principles; but, in conſequence of this union, chemical effects are *animalized*, or become *animal* indications; and animal operations can no more be aſcribed to chemical powers, excluſively, than the latter can be referred to the laws of animal life. The matter of heat, for example, is an eſſential and operative power, both in living and in dead bodies; but in its union with living animals, it loſes a part of its chemical properties; it unites and accommodates itſelf to the nature of the living ſubſtance; in a word, it becomes *animal heat*. Electricity is, without diſpute, one of the moſt powerful ſtimulants of the animal frame; but as ſoon as it unites with the living power, it is diveſted of ſome portion of its natural properties, forms a new relation and connection with organized nature, by means of which it follows the laws of animo-chemical nature, and becomes *animal* electricity. Hence it is, that in ſome caſes animal heat and animal electricity deviate from the common laws of Nature, by virtue of their union with animal ſubſtances. For example, it is a common law of Nature, that a body ſhall finally acquire an equal degree of heat with the atmoſphere in which it is placed; but in living beings, this law is expoſed to many exceptions; for, in warm-blooded animals, the temperature of the animal is much cooler than the atmoſpheric air, when it riſes to a high degree of heat; and much warmer when the atmoſpheric air is cold. Thus human beings are as capable of living under the burning ſuns of Senegal, which is ſufficiently hot to boil ſpirits, as in the pinching cold of Hudſon's Bay, by which both ſpirits and mercury are frozen; and the medium heat of the internal parts of the body remains at about 98 degrees of Fahrenheit's thermometer, though expoſed to theſe two extremes of heat and cold. Thus the vital power, and the laws of animal Nature dependent upon it, regulate the influence of the caloric, or matter of heat, in ſuch a manner as to maintain animal heat in its medium ſtate.

We ſubſcribe to the ſentiments of the great Hufeland, as moſt conſonant with appearances, viz. that although the powers of animated matter may be influenced in many reſpects by mechanic or chemical properties, yet they are not to be confounded with them; ſince their laws of action, and their various operations, cannot be explained by any of the principles of mechanics or chemiftry which are hitherto known, and appear very diftinct from the laws which govern inactive matter.

The phænomena of life (as the fame author has juftly remarked) may be reduced to two heads. They evidence a *susceptibility* of ftimulants, and fpecific mixtures, changes, and formations of organized matter; fo that the two principal characteriftics of the vital powers confift in the capacity of a living being to receive impreffions from ftimuli, and re-act upon them according to its own laws, which are diftinct from thofe of chemiftry or mechanics; and, fecondly, in the power of organized bodies to change ufual affinities and combinations in fuch a manner, that very different affinities and combinations are produced, by which new products, forms, and creations are rendered poffible; of the exiftence of which, we can find no traces throughout Nature but in living beings.

The arguments of the Chemical Sect, however fpecious and conclufive they may appear upon a fuperficial view, cannot bear the teft of critical examination, and will prove unfatisfactory to every impartial mind; as the following Obfervations will, we truft, fully evince.

1. A palpable Induction takes place in their manner of reafoning; for, becaufe the diverfities in matter in general depends upon a difference in its combinations, they infer that the indications of a particular property in bodies, and, confequently, their nature, is alone to be afcribed to the different proportions in the mixture of their component parts; and this being a common law of Nature, muft alfo be extended to organized bodies; fo that every appearance in thefe is equally to be afcribed to different proportions in the combinations of animal matter, and their mutual attractions to each other. This they unphilofophically confider as an univerfal rule, and thus apply it to living beings. But the univerfality of this rule is very doubtful, particularly when applied to organized bodies; and, confequently, as the propofition cannot be demonftrated, it cannot be with fafety applied to living beings.

2. Their arguments are obvioufly founded on a *petitio principii.* Experience, they fay, teaches us that a fpecies of matter called animal matter exifts, and poffeffes life; but the grand queftion whether this organized matter poffeffes life *in itself*; that is, alone proceeding from its form and combinations, is paffed over in profound filence. But as a judicious author obferves, fince there is not in all Nature any fpecies of matter known, to which the character of *vitality* belongs, we cannot be authorized to afcribe this vitality to any chemical action, or combination of thefe dead materials. That matter is capable of various degrees of excellence is allowed, but it does not follow neceffarily neceſſarily from hence that this excellence aſcends up to vitality itſelf; much leſs that the vital powers are the neceſſary conſequence of this exaltation of matter alone. This is merely hypothetical, unſupported by the ſmalleſt proof. The extravagance of the ſuppoſition becomes yet ſtronger by the conſideration that although chemiſts, and moreover Nature itſelf, ſo frequently mix very different materials, expoſed to innumerable attractions, and the moſt oppoſite actions both of mechanical and chemical laws, yet they have never produced the leaſt tokens either of organization or of vitality.

3. They explain a propoſition by itſelf, that is, they argue in a circle. According to their doctrine, the chief cauſe of form and combination of organized matter conſiſts in the form and combination of organizing materials. For if figure and organization are only an indication and operation of this matter, we are not to conſider theſe indications and operations as proceeding from figure or organization; for it is an abſurdity to ſuppoſe any thing to be indicative and operative, and at the ſame time to be the cauſe of theſe effects. It is clear to a demonſtration that we muſt have recourſe to ſome other cauſe. Nor can this cauſe be diſcovered in the original difference in the conſtituent parts of organized matter, or in any ſpecific mixture; for theſe, as well as form, are mere *indications*: Therefore the cauſe of vitality, in animal matter, cannot be explained by its form and organization, but it muſt ariſe from ſomething eſſentially different.

4. Though it is acknowledged that ſpecific combinations and organizations, and ſpecific powers, acquire new relations to each other, and that a change in the firſt is followed by a correſponding change in the other; yet we are not entitled to conclude from hence, that the former is the cauſe, and the latter the effect. Such combinations may only be conſidered as a *conditio sine qua non.* A muſician is able to produce various tones from a flute, a violin, or a trumpet; but are we to infer from hence, that this power reſides ſimply and ſolely in the inſtrument? The inſtrument, without the aſſiſtance of foreign aid, is not able to utter the ſmalleſt ſound; in like manner does ſomething ſuperadded to combinations and organizations become neceſſary to produce the effects which the Chemical Sect aſcribe ſolely to theſe.

5. Again, this ſect does not make a ſufficient diſtinction between the firſt principle of life, and the intermediate cauſes of the appearances of vitality in organized beings. They univerſally confound the cauſe of vitality with the cauſes of its operations, and this appears to us to be the ſource of their miſconceptions and erroneous doctrines. They reſt ſatisfied with aſcribing cribing the whole to organization, without examining in what manner this arrangement of parts, and consequent form, was produced; or what was the original cause. They do not reflect, that the organization and combinations which indicate such wonderful powers, are *effects*, of which the principle of life or vital power, is the first and efficient *cause*; that this last is *active*, and that the inanimate matter upon which it operates is *passive*; so that organic matter, and the organs produced from it, are the instruments of its power, and subject to its laws.

6. Were we to acknowledge that the truth of their hypothesis was fully established, we should not advance a single step in our knowledge of organic powers. It is true, the advocates for the chemical theory are fond of exalting matter; speak much of an affinity approaching to intelligence in the matter composing organized bodies, of specific organized affinities, of animal crystallizations, by which they attempt to explain the theory of propagation and formation of organs; yet, placing the phænomena of animation in this point of view, notwithstanding its novelty, does not enable us to penetrate farther into the subject than any preceding theory: For all these chemical principles have certainly, in their effects upon *dead* matter, decisive tokens; but when applied to the *animal* body, they become unintelligible, and in place of the promised elucidation, present us only with a new phraseology, and new words that explain nothing. We knew before, that matter may possess various degrees of excellence; that mineral substances pass over into vegetable, and these into animal; that in organized Nature the solids are composed of fluids; and that a drop of moisture is the origin of animated beings. The *modus quo* alone remained unexplored. We were ignorant in what manner the bones, muscles, nerves, vessels, so very different in their natures and contextures, could be formed out of the same mass of fluids, the blood. But their chemical explanations leave us as much in the dark as we were before. In what are we advanced by being informed that these various parts are generated by some peculiar relationship or affinity; these fluids *crystallize* themselves into bones, sinews, &c. &c.? The cool Inquirer will not be satisfied with these vague statements, he will persist in demanding, by what power are inanimate bodies, of various natures, united to animal substance? and by what power are they changed into those various component parts which constitute the different organs? How is it that these, which in every other union still remain dead and inanimate, assume, in animal substances, a character totally different, with totally different indications? Surely every unprejudiced person, who duly duly confiders thefe facts, will feel himfelf compelled to acknowledge, that a different principle exifts in organized bodies, which, by its union with inanimate matter, is productive of fpecific organic combinations, and, with thefe, the different phænomena of organic bodies.

[ To be continued. ]

